# Strontium Ranelate Ameliorates Intervertebral Disc Degeneration via Regulating TGF-β1/NF-κB Axis

**DOI:** 10.7150/ijms.86665

**Published:** 2023-10-07

**Authors:** Ruping Sun, Jian Zhu, Kaiqiang Sun, Lu Gao, Bing Zheng, Jiangang Shi

**Affiliations:** 1 School of Health Science and Engineering, University of Shanghai for Science and Technology, No. 516 Jungong Road, Shanghai 200093, China; 2 Department of Orthopedics, Changzheng Hospital, Naval Medical University, No.415 Fengyang Road, Shanghai 200003, China.; 3 Department of Orthopaedic Surgery, Naval Medical Center, Naval Medical University, Shanghai 200433, China.; 4 Department of Department of Physiology, Naval Medical University, Shanghai 200433, China.

**Keywords:** Strontium Ranelate, IVDD, Targeted treatment, TGF-β1/NF-κB Axis

## Abstract

Intervertebral disc degeneration (IVDD) is a prevalent and debilitating condition characterized by chronic back pain and reduced quality of life. Strontium ranelate (SRR) is a compound traditionally used for treating osteoporosis via activating TGF-β1 signaling pathway. Recent studies have proved the anti-inflammatory effect of SRR on chondrocytes. Although the exact mechanism of IVDD remains unclear, accumulating evidences have emphasized the involvement of multifactorial pathogenesis including inflammation, oxidative stress damage, and etc. However, the biological effect of SRR on IVDD and its molecular mechanism has not been investigated. Firstly, this study proved the decreased expression of Transforming Growth Factor-beta 1(TGF-β1) in degenerated human intervertebral disc tissues. Subsequently, we confirmed for the first time that SRR could promote cell proliferation, mitigate inflammation and oxidative stress in human nucleus pulposus cells *in vitro* via increasing the expression of TGF-β1 and suppressing the Nuclear Factor Kappa-Light-Chain-Enhancer of Activated B Cells (NF-κB) pathway. The molecular docking result proved the interaction between SRR and TGF-β1 protein. To further verify this interaction, gain- and loss- of function experiments were conducted. We discovered that both TGF-β1 knockdown and overexpression influenced the activation of the NF-κB pathway. Taken together, SRR could mitigate IL-1β induced-cell dysfunction in human nucleus pulposus cells by regulating TGF-β1/NF-κB axis *in vitro*. Finally, the *in vivo* therapeutic effect of SRR on IVDD was confirmed. Our findings may contribute to the understanding of the complex interplay between inflammation and degenerative processes in the intervertebral disc and provide valuable insights into the development of targeted treatment-based therapeutics for IVDD.

## Introduction

Intervertebral disc degeneration (IVDD) is a highly prevalent musculoskeletal disorder that affects millions of individuals worldwide, leading to chronic back pain, reduced quality of life, and substantial economic burden^1^,^2^. The age-related degenerative process of the intervertebral disc involves alterations in its anatomical structure, biomechanical properties, and biochemical composition, which ultimately result in functional impairment and clinical manifestations^3^.At the cellular level, cell proliferation is an essential process associated with tissue regeneration and repair. Both Proliferating Cell Nuclear Antigen (PCNA) and Ki-67 are established markers for cell proliferation. Furthermore, IVDD is characterized by extracellular matrix (ECM) degeneration. Specifically, a decrease in aggrecan and type II collagen, which are positive markers for ECM integrity, is observed. Conversely, an increase in A Disintegrin And Metalloproteinase with Thrombospondin Motifs 5 (ADAMTS5) and Matrix Metalloproteinase 3 (MMP3), serving as negative markers indicative of ECM degradation, is noted in IVDD.

Epidemiological studies have demonstrated a high prevalence of IVDD, affecting over 80% of the population aged 40 years and above. The progressive nature of IVDD often results in disability and a decrease in productivity, placing a significant burden on healthcare systems and societies^4^. Among the multiple factors contributing to the progression of IVDD are oxidative stress and inflammation^5^-^8^. At the molecular level, Western blot analysis has demonstrated that NADPH oxidase 2 and NADPH oxidase 4 (NOX2/4), serving as positive markers for cellular oxidative stress, were upregulated in response to Interleukin-1 beta (IL-1β). On the other hand, Superoxide Dismutase 1 (SOD1) and Heme oxygenase-1 (HO-1), both recognized as positive markers for antioxidative defense, exhibited decreased expression. Additionally, we explored the impact of SRR on the IL-1β-induced inflammatory response. Markers such as Cyclooxygenase-2 (COX-2), inducible Nitric Oxide Synthase (iNOS), Interleukin-6 (IL-6), Nitrite, Prostaglandin E2 (PGE2), and Tumor Necrosis Factor-alpha (TNF-α) have been identified as established indicators of inflammation.

IVDD originates from a multifaceted combination of genetic predispositions, mechanical stressors, and environmental influences. Among these, inflammation is progressively gaining recognition as a critical orchestrator, steering the path towards degenerative processes^5^,^6^. IL-1βis a pro-inflammatory cytokine produced by activated macrophages and is involved in various cellular activities, including cell proliferation, differentiation, and apoptosis. As a critical mediator in the inflammatory response, IL-1β has been implicated in a multitude of pathological conditions and is closely related to inflammatory processes and oxidative stress. Within the context of musculoskeletal disorders, such as IVDD, IL-1β contributes to the inflammatory milieu, exacerbating tissue damage and promoting degenerative changes. This cytokine has been consistently linked with increased ECM degradation, highlighting its role in modulating disc health. Given the emphasis on inflammation in the pathogenesis of IVDD, understanding the role and regulation of cytokines like IL-1β becomes pivotal in deciphering disease mechanisms and devising therapeutic strategies. Extensive studies have shown that multiple pathogenic factors, including inflammation, oxidative stress, and apoptosis, cooperatively contribute to the progression of IVDD^7^,^8^. However, despite substantial efforts, the precise pathogenic mechanisms underlying IVDD remain to be fully elucidated. Notably, Transforming Growth Factor-β1 (TGF-β1) has assumed center stage as a vital regulatory factor. TGF-β1's critical role in IVDD has been substantiated by a multitude of research. For instance, it has been shown to inhibit disc cell apoptosis and promote extracellular matrix synthesis, thereby maintaining disc homeostasis and delaying IVDD progression^7^-^9^. Furthermore, TGF-β1 was found to protect intervertebral discs from degeneration under excessive mechanical loading conditions, suggesting its role in countering mechanical stress-induced IVDD^10^. In addition, TGF-β1 has been reported to mitigate inflammation and oxidative stress in IVDD, underlining its protective role^11^,^12^. Given the extensive influence of TGF-β1 in IVDD pathogenesis, we hypothesize that molecular treatments aimed at enhancing TGF-β1 could potentially serve as curative methods for IVDD. On a related note, agents that have been shown to target inflammation and oxidative stress, such as Strontium ranelate (SRR), may also hold promise for IVDD treatment.

Strontium ranelate (SRR) is an established therapeutic agent for osteoporosis, known to promote bone formation and inhibit bone resorption^13^. Originating as a primary treatment for osteoporosis, its mechanism of action is twofold; promoting bone formation and attenuating bone resorption, thus providing a balanced approach to bone health^14^. The unique dual action of SRR on bone metabolism is attributed to its ability to influence osteoblasts (bone-forming cells) and osteoclasts (bone-resorbing cells) activities^14^. This equilibrium not only enhances bone mineral density but also reduces the risk of fractures, a frequent complication of osteoporosis^15^. Transitioning to the context of intervertebral discs, the health and integrity of the adjacent vertebral bones play a significant role in determining disc health. Bone quality can impact the mechanical stresses experienced by the discs, potentially influencing the risk and progression of IVDD. Given the close anatomical and functional relationship between vertebrae and intervertebral discs, treatments that can benefit bone health could conceivably have implications for disc health as well. Moreover, as IVDD is often accompanied by changes in the vertebral endplates and subchondral bone, the role of agents like SRR, which directly target bone metabolism, becomes of paramount importance^16^.Recent evidence has additionally highlighted its potent anti-inflammatory properties, lending credibility to its potential application in conditions characterized by inflammation^17^. Notably, SRR has been shown to suppress inflammatory responses by inhibiting the production of pro-inflammatory cytokines^17^,^18^. Furthermore, it has demonstrated protective effects against oxidative stress-induced cell damage^19^,^20^ and has been reported to modulate apoptosis in various cell types^21^. However, it is important to acknowledge that the use of SRR also has some drawbacks. For instance, it has been associated with an increased risk of venous thromboembolism and serious skin reactions^22^,^23^. Therefore, the potential benefits of SRR in IVDD treatment should be carefully weighed against its potential adverse effects.

The nuclear factor-kappa B (NF-κB) signaling pathway is implicated in the regulation of inflammation, cell proliferation, and ECM homeostasis in the intervertebral disc^24^,^25^. Dysregulation of the NF-κB pathway has been associated with the severity of IVDD, as reflected by increased ECM degradation, reduced disc cell viability, and enhanced inflammatory response. Therefore, modulating the NF-κB pathway may offer therapeutic potential in the context of IVDD. Intriguingly, SRR has been implicated in the regulation of the NF-κB pathway. Recent studies have unveiled the potential of SRR to inhibit the activation of the NF-κB signaling pathway^26^,^27^.These findings highlight the potential of SRR as a potent modulator of the NF-κB pathway, suggesting its therapeutic utility in the treatment of IVDD. However, whether these protective effects above of SRR are also exist in IVDD, how SRR affects the biological behavior of human nucleus pulposus (HNP) cells, and the exact molecular mechanism of the effect of SRR on nucleus pulposus (NP) cells remain elusive.

The current study aims to 1) investigate the shifting expression patterns of TGF-β1; 2) reveal the biological impact of SRR on NP cells and the underlying molecular mechanisms *in vitro*; 3) substantiate the *in vivo* effectiveness of SRR as a treatment for IVDD. The findings of our study may deepen the understanding of IVDD pathogenesis and explore novel therapeutic strategies for treating IVDD.

## 2. Materials and Methods

### 2.1. Acquisition and Patient Samples

The present study received approval from the Ethics Committee of Shanghai Changzheng Hospital, Naval Medical University, and written informed consent was obtained from all participating patients. The NP tissues were harvested from patients undergoing lumbar discectomy surgery. Based on preoperative MRI Pfirrmann grades, NP tissues were classified into three groups: non-degenerative or slight-degenerative group (Grade I or II), moderate degeneration group (Grade III), and severe degeneration group (Grade IV or V). All experiments involving human NP (HNP) samples in this study adhered to the principles outlined in the Helsinki Declaration (World Medical Association, 2014).

### 2.2. HNP Cells Isolation, Culture, and Identification

The isolation and culture method for HNP cells was adapted from our previous study^28^. Briefly, intraoperatively harvested NP tissues were stored in 0.9% sodium chloride solution and promptly transferred to an ultra-clean table. The NP tissues were washed three times with sterile phosphate-buffered saline (PBS, G4202, Servicebio, China), followed by a digestion step using 0.2% collagenase type II (Invitrogen, USA) mixed with complete medium (0.1% FBS+1% penicillin-streptomycin mix + DMEM/F-12 medium) for one hour at 37.5°C under a shaker (70 rpm). After centrifugation at 1200 rpm for 5 minutes, the isolated HNP cells were resuspended in complete medium and seeded in T25 culture flasks in an incubator (37°C, 5% CO2). Approximately five days after isolation, the spindle-shaped cells adhering to the flask bottom were identified as passage 0 HNP cells. HNP cells were grown to 80% confluency before being used in subsequent experiments.

### 2.3. TUNEL Assay

Following fixation with 4% paraformaldehyde for 30 minutes, the disc samples were washed with PBS 3-4 times. Next, NP cells were permeabilized using 0.3% Triton for 5-10 minutes. The samples were then incubated with a fluorescein (FITC) TUNEL Cell Apoptosis Detection Kit (G1501-100T, Servicebio, Wuhan, China) for 1 hour. Subsequently, the nuclei were stained with 2-(4-amidinophenyl)-1H-indole-6-carboxamidine (DAPI) solution in a dark environment. Finally, images of apoptotic cells were captured using a fluorescence microscope (Olympus, Japan).

### 2.4. Immunohistochemical (IHC) Analysis

The IHC analysis was carried out as follows: Briefly, HNP tissue sections embedded in paraffin were cut into 15 μm sections. These sections were then deparaffinized with environment-friendly de-paraffin liquid (G1128, Servicebio, China) and dehydrated using gradient alcohol. Membrane-breaking solution (G1204, Servicebio, China) was applied according to the protocols. The sections were subsequently incubated with 3% BSA for 25 min to block endogenous peroxidase, followed by overnight incubation at 4°C with primary antibodies against IL-1β (#AF4006, Affinity, China, 1:200), p65 (#AF5006, Affinity, China, 1:200), and TGF-β1 (#AF1027, Affinity, China, 1:200). The next day, sections were incubated with HRP-conjugated Goat Anti-Rabbit IgG H&L (511,203, ZENBIO, China, 1:300) for 1 h, and counterstaining was performed with hematoxylin solution for 5 min. Images of stained sections were acquired using a light microscope (BX43, Olympus, Japan).

### 2.5. Real-Time Quantitative PCR (qRT-PCR)

The qRT-PCR procedure was conducted as previously described^29^. Briefly, total RNA was extracted using the Rapture Universal RNA Plus Kit (R4013-02, Magen, China) according to the manufacturer's instructions. The purified total RNA was then reverse-transcribed to cDNA, which was subsequently quantified using the ChamQ Universal SYBR qPCR Master Mix (Q711-03, Vazyme, China) in a StepOnePlus PCR System (Applied Biosystems, USA). The GAPDH level was employed for normalization. The expression of the target genes relative to GAPDH was quantified using the 2^^-ΔΔCt^ method and reported as the fold change.

### 2.6. CCK-8 Assay

For the CCK-8 assay, HNP cells were seeded in a 96-well plate at a density of 4 × 10^3^ cells per well and cultured for 24 hours. The cells were then treated with various concentrations of SRR (0, 0.125, 0.25, 0.5, 1, 2, 5, 10 mM) for an additional day. Following this, the HNP cells were treated with 100 μl of CCK-8 detection solution (10 μl CCK-8 solution and 90 μl DMEM/F-12 medium) and cultured for 1 hour. The absorbance was measured at a wavelength of 450 nm using a microplate reader.

### 2.7. Immunofluorescence Analysis

HNP cells were immunofluorescently stained for aggrecan (#DF7561, Affinity, China, 1:200), type II collagen (#AF0135, Affinity, China, 1:200), MMP3 (#AF0217, Affinity, China, 1:200), Ki-67 (#AF0198, Affinity, China, 1:200), p65 (#AF5006, Affinity, China, 1:200), and TGF-β1 (#AF1027, Affinity, China, 1:200), following a previously described protocol^28^. Briefly, samples were fixed with 4% PFA for 25 minutes and permeabilized with 0.1% vol/vol Triton X-100 for 10 minutes. Samples were then blocked with 5% BSA at room temperature for 60 minutes. Following this, the samples were incubated with the appropriate primary antibodies at 4°C overnight. The HNP cells were washed three times with cold PBS and incubated with a 1:500 dilution of a secondary antibody (550,076, Zen Bio, China) at room temperature for 60 minutes. Nuclei visualization was performed using DAPI. Finally, HNP cells were sealed with an anti-fluorescence quencher (G1401, Servicebio, China) and observed under a fluorescence microscope (DS-Ri2, Nikon, Japan).

### 2.8. Cell Proliferation Assay Using BeyoClick™ EdU Cell Proliferation Kit with Alexa Fluor 488

Cell proliferation was assessed using the BeyoClick™ EdU Cell Proliferation Kit with Alexa Fluor 488 (Beyotime) according to the manufacturer's instructions. HNP cells were seeded into 24-well plates at a density of 1×10^4^ cells per well and treated with SRR at different concentrations (0, 1, 10, 100, and 1000 μM) for 48 hours. After treatment, the cells were incubated with 10 μM EdU for an additional 2 hours at 37°C. The cells were then fixed with 4% paraformaldehyde, permeabilized with 0.5% Triton X-100, and stained with the Click reaction mixture containing Alexa Fluor 488 azide. The nuclei were counterstained with DAPI, and the EdU-positive cells were visualized under a fluorescence microscope. The percentage of EdU-positive cells was calculated by counting the number of EdU-positive cells relative to the total number of DAPI-stained cells in five random fields per well. The experiments were performed in triplicate, and the data were presented as the mean ± standard deviation (SD).

### 2.9. Western Blot (WB) Analysis

The Western Blot process has been previously described in study 29. Briefly, total proteins from different groups were extracted using a whole cell lysis assay kit (KGP250/KGP2100, KeyGEN BioTech, China) following the manufacturer's instructions. Protein concentration was quantified using a BCA Protein Assay Kit, and protein solutions with equal concentrations were prepared with Omni-Easy™ Protein Sample Loading Buffer (LT101S, Epizyme Biomedical Technology Co., Ltd, China). Equal amounts of total protein (80 μg) per well were separated by 10% SDS-PAGE electrophoresis. Electrophoresis was conducted at 250 V for 30 minutes, and proteins were then transferred to a 0.45 μm pore size PVDF membrane (IPVH00010, Millipore, USA). Proteins were incubated with protein-free quick block solution (G2052, Servicebio, China), followed by incubation with primary antibodies against NOX2 (381293, Zen Bio, China, 1:1500), SOD1 (10269-1-AP, Protein Tech, China, 1:5000), HO1 (10701-1-AP, Protein Tech, China, 1:5000), TNF-α (17590-1-AP, Protein Tech, China, 1:1500), IL-1β (16806-1-AP, Protein Tech, China, 1:1500), iNOS (22226-1-AP, Protein Tech, China, 1:1000), IκB-α (10268-1-AP, Protein Tech, China, 1:3000), TGF-β1 (AF1027, Affinity, China, 1:1500), PCNA (BF0704, Affinity, China, 1:1500), NOX4 (DF6924, Affinity, China, 1:1500), p-IκB-α (#2859, Cell Signaling Technology, USA, 1:1000), IL-6 (DF6087, Affinity, China, 1:1500), P65 (66535-1-Ig, Protein Tech, China, 1:1500), p-P65 (310012, Zen Bio, China, 1:1000), Actin (GB11001, Servicebio, China, 1:1500), Histone H3 (GB11102, Servicebio, China, 1:500), ACAN (DF7561, Affinity, China, 1:1500), ADAMTS5 (DF13268, Affinity, China, 1:1500), Col2 (AF0135, Affinity, China, 1:1500), MMP3 (AF0217, Affinity, China, 1:1500), and GAPDH (GB15002,Servicebio, China,1: 2000), at 4°C overnight. After washing with TBST three times, proteins were incubated with HRP-conjugated Goat Anti-Rabbit IgG H&L (511,203, ZENBIO, China, 1:3000) or Goat Anti-Mouse IgG (1:3000) for 2 hours at room temperature. Following washing with TBST, immunoreactive bands were visualized and detected using the Omni-ECL™ Femto Light Chemiluminescence Kit as per the manufacturer's instructions (SQ201L, Epizyme Biomedical Technology Co.,Ltd, China). The bands were then exposed to X-ray film and developed using an Automatic Chemiluminescence/-Fluorescence Image Analysis System (5200, Tanon, China).

### 2.10. Enzyme-Linked Immunosorbent (ELISA) Assay

To obtain the conditioned media for cytokine array analysis, NP cells were subjected to different treatments (Control, IL-1β, IL-1β+SRR) for 24 hours without changing the culture medium. Subsequently, the conditioned media from each group were collected and centrifuged at 5000 rpm and 4°C for 15 minutes. The supernatants were stored at -80°C separately until use and were diluted with appropriate standard diluents before measurement. The levels of ADAMTS5 (DY2198-05, R&D Systems, USA), collagen-II (F15215, Westang, China), MMP-3 (F01870, Westang, China), Aggrecan (DY1220, R&D Systems, USA), MDA (F01963, Westang, China), SOD1 (F11502, Westang, China), TNF-α (F02810, Westang, China), IL-6 (F01310, Westang, China), Nitrite (KGE001, R&D Systems, USA), and PGE2 (F02290, Westang, China) in the supernatants were determined using Enzyme-Linked Immunosorbent Assay (ELISA) kits. The optical density was measured at a wavelength of 450 nm.

### 2.11. Lipid peroxidation assay

The level of lipid peroxidation, an indicator of oxidative stress, was assessed using the BODIPY™ 581/591 C11 sensor (D3861; Invitrogen™) according to the manufacturer's instructions. Briefly, HNP cells were incubated with 2 μM BODIPY™ 581/591 C11 for 1 hour, followed by washing twice with sterile cold PBS. Subsequently, the imaging slides were fixed with 4% paraformaldehyde and sealed with an anti-fluorescence quencher (G1401, Servicebio, China). The change in lipid peroxidation was standardized and represented as the ratio of green fluorescence (oxidative state) to red fluorescence (non-oxidative state).

### 2.12. The Detection of mitochondrial membrane potential

The functional status of mitochondria was assessed using a JC-1 Staining Kit (C2003S, Beyotime, China) following the manufacturer's instructions. Briefly, after washing with cold PBS, HNP cells were treated with 1 mL of JC-1 staining solution and incubated at 37°C with 5% CO2 for 20 minutes. Subsequently, 2 mL of complete medium (0.1% FBS + 1% penicillin-streptomycin mix + DMEM/F-12 medium) was added to the HNP cells. The fluorescence of JC-1 was detected using a fluorescence microscope (Olympus, Japan).

### 2.13. Superoxide assay

Superoxide levels were assessed using a Superoxide Assay Kit (S0060, Beyotime, China) following the manufacturer's instructions. Briefly, the working solution for each detection was prepared in the following proportions: 200 μl superoxide detection buffer, 10 μl WST-1 solution, and 2 μl catalase. A volume of 200 μl superoxide test solution was added to each well and incubated at 37°C for 3 minutes. Absorbance was measured at a wavelength of 450 nm using a microplate reader.

### 2.14. Molecular docking

Molecular docking was performed using the 3D structure of the TGF-β1 protein obtained from the RCSB database (PDB ID: 5VQP). Prior to docking, the missing structures were repaired using Chimera 1.15. The 3D structure of the small molecule Strontium ranelate was downloaded from the PubChem database (PubChem CID: 3052774) and energy-minimized under the MMFF94 force field using AVOGADR 1.2.0. AutoDock Vina 1.1.2 software was employed for molecular docking in this study. Before docking commenced, the receptor protein structure was processed and hydrogenated using the academic open-source version of PyMol. Subsequently, ADFRsuite 1.0 was used to convert all processed small molecules and receptor proteins into the PDBQT format required for docking with AutoDock Vina 1.1.2. Prior to docking, a box was constructed with suitable X, Y, and Z dimensions, centered around the protein's centroid (protein center coordinates are shown in Table 1) to fully encapsulate the entire protein During docking, the grid box and processed protein and small molecule PDBQT files were used as input files for Vina. The exhaustiveness of the global search was set to 32, while other parameters remained at default settings. The highest-scoring docking conformation was considered the binding conformation, and the docking results were visualized and analyzed using the academic open-source version of PyMol.

### 2.15. Small interfering RNA (siRNA) transfection

To investigate the role of TGF-β1 in our study, small interfering RNA (siRNA) targeting TGF-β1 was employed to silence its expression. The small interfering RNA for TGF-β1 (siRNA-TGF-β1) and a non-targeting control siRNA were synthesized and purchased from a commercial supplier.HNP cells were seeded into 6-well plates and cultured until they reached 60-70% confluence. Transfection was performed using Lipofectamine 3000 reagent (Invitrogen, USA) according to the manufacturer's instructions. Briefly, the siRNA-TGF-β1 or control siRNA were diluted in Opti-MEM medium (Gibco, USA) and mixed with Lipofectamine 3000. The mixture was incubated at room temperature for 15-20 minutes to allow complex formation. The complexes were then added to the NP cells and incubated for 48 hours at 37°C in a humidified atmosphere with 5% CO_2_. Post-transfection, the efficiency of TGF-β1 knockdown was assessed using real-time quantitative PCR (qRT-PCR) and Western blot analysis. The transfected cells were subsequently used for further experiments to elucidate the functional role of TGF-β1 in our experimental context.

### 2.16. Plasmid Transfection

To overexpress TGF-β1 in HNP cells, a plasmid carrying the TGF-β1 gene was utilized. The TGF-β1 plasmid and an empty control vector were obtained from a commercial supplier. HNP cells were seeded into 6-well plates and cultured until they reached 60-70% confluence. Transfection was performed using Lipofectamine 3000 reagent (Invitrogen, USA) following the manufacturer's instructions. Briefly, the TGF-β1 plasmid or control vector were diluted in Opti-MEM medium (Gibco, USA) and combined with Lipofectamine 3000. The mixture was incubated at room temperature for 15-20 minutes to facilitate complex formation. The complexes were then added to the NP cells and incubated for 48 hours at 37°C in a humidified atmosphere with 5% CO_2_. After transfection, the overexpression efficiency of TGF-β1 was assessed using real-time quantitative PCR (qRT-PCR) and Western blot analysis. The transfected cells were subsequently used in further experiments to elucidate the functional consequences of TGF-β1 overexpression in our experimental setup.

### 2.17. Safranin O-Fast Green Staining

Intervertebral disc tissue samples were fixed in 4% paraformaldehyde for 48 hours at room temperature, dehydrated in a graded series of ethanol, and then embedded in paraffin. Tissue sections of 5 μm thickness were cut using a microtome.For Safranin O-Fast Green staining, sections were deparaffinized in xylene and rehydrated through a graded ethanol series to distilled water. The sections were stained in Weigert's iron hematoxylin solution for 8 minutes, washed in running tap water for 10 minutes, and then rinsed in distilled water. The sections were stained with 0.02% Fast Green solution for 5 minutes, rinsed quickly in 1% acetic acid solution, and then stained in 0.1% Safranin O solution for 5 minutes. After staining, the sections were quickly dehydrated in 95% ethanol, absolute ethanol, and xylene, each for 2 minutes, and then mounted with a synthetic resin.

### 2.18. In vivo Mouse Imaging via Magnetic Resonance Imaging (MRI)

*In vivo* mice spine imaging was executed using a 7.0 T magnetic resonance imaging (MRI) system (Bruker BioSpec 16 US/Pharmascan, Germany), specifically tailored for small animals. The protocol encompassed a variety of sequences, including T1-weighted, proton density-weighted, FLAIR, and T2-weighted gradient echo sequence with echo time/repetition time parameters set at 27/3000 ms. T2-weighted images were thoroughly analyzed and assessed in both sagittal and axial planes using the RadiAnt DICOM Viewer software (V4.6.9, Medxant, Poznan, Poland). The quantification of intervertebral discs displaying a hypointense signal was carried out manually by a researcher who was blind to the experimental conditions.

### 2.19. Statistical analysis

All experiments in this study were conducted independently at least three times. Data were analyzed using SPSS version 26.0 software (IBM, Armonk, NY, USA). Continuous variables were presented as mean values ± standard deviation (SD), while categorical variables were expressed as percentages (%). Comparisons of mean values or data distribution for continuous variables were performed using unpaired two-tailed Student's t-test or Mann-Whitney U test, as appropriate. Categorical variables were compared using the χ2 (chi-square) test or Fisher's exact test, as appropriate. A P value of <0.05 was considered to indicate statistical significance.

## 3. Results

### 3.1. Activation of NF-κB and Decreased Expression of TGF-β1 in Degenerated Human Intervertebral Disc Tissues

**Figure 1:**
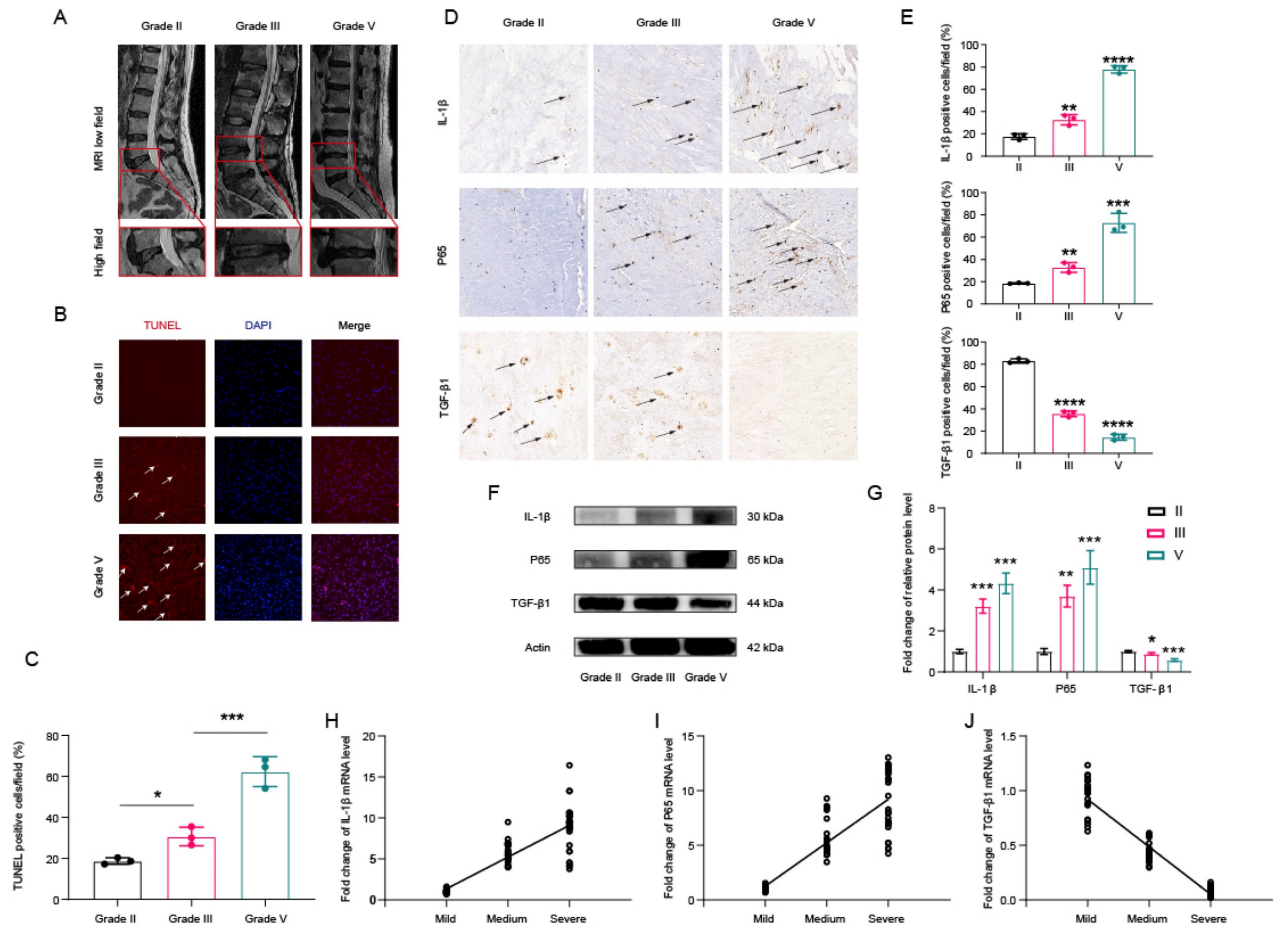
** The protein expression of TGF-β1 and p65 in human NP tissues.** (A) The human NP tissues were divided into mild group (Grade II), moderate degeneration group (Grade III) or severe degeneration group (Grade IV or V) according to the Pfirrmann grades. (B) The apoptotic NP cells (red) in intervertebral disc between GradeII, III and Grade V were visualized via TUNEL staining and the nuclei were stained with DAPI. Scar bar = 300 μm. (C) Three randomized versions were selected, and TUNEL staining-positive cells were quantified via the amount of red fluorescence. (D) Immunohistochemical results were used to examine the protein levels of IL-1β, p65, and TGF-β1 in the human NP tissue with Grade II, III and Grade IV. (E) Three versions were randomly selected, and the stained cells were quantified separately. (F,G) Protein bands and quantification of expression levels of IL-1β,p65, andTGF-β1. Scar bar = 100 μm. (H-J) Changes in the expression of IL-1β, p65, and TGF-β1 were further confirmed by qPCR, which was consistent with the above results. ∗p < 0:05, ∗∗p < 0:01, ∗∗∗p < 0:001, ∗∗∗∗p < 0:0001.

HNP tissues were categorized into mild (Grade II), moderate (Grade III), or severe (Grade IV or V) degeneration groups according to Pfirrmann grades based on pre-oeprative MRI image (Figure 1A)^30^. As depicted in Figure 1B, degenerated disc tissues exhibited higher numbers of TUNEL-positive cells. The expression of TUNEL in degenerated disc tissues was quantified and standardized, as shown in Figure 1C. Furthermore, immunohistochemical analyses revealed a significantly higher proportion of IL-1β- and NF-κB-positive cells, along with a lower proportion of TGF-β1-positive cells in HNP tissues with degenerated intervertebral discs (Figures 1D and 1E). Western blot analyses also suggested that Grade IV intervertebral disc tissue samples exhibited typical IVDD features, including increased expression of IL-1β and p65, as well as decreased expression of TGF-β1 (Figures 1F and 1G). Subsequent qPCR analyses demonstrated that the mRNA levels of IL-1β and p65 increased with the progression of disc degeneration, while the mRNA levels of TGF-β1 decreased accordingly (Figures 1H and 1J). Collectively, these findings indicate that lower levels of TGF-β1 may be associated with greater severity of IVDD.

### 3.2. SRR Could Enhance HNP Cell Viability and Promote NP Cell Proliferation

**Figure 2:**
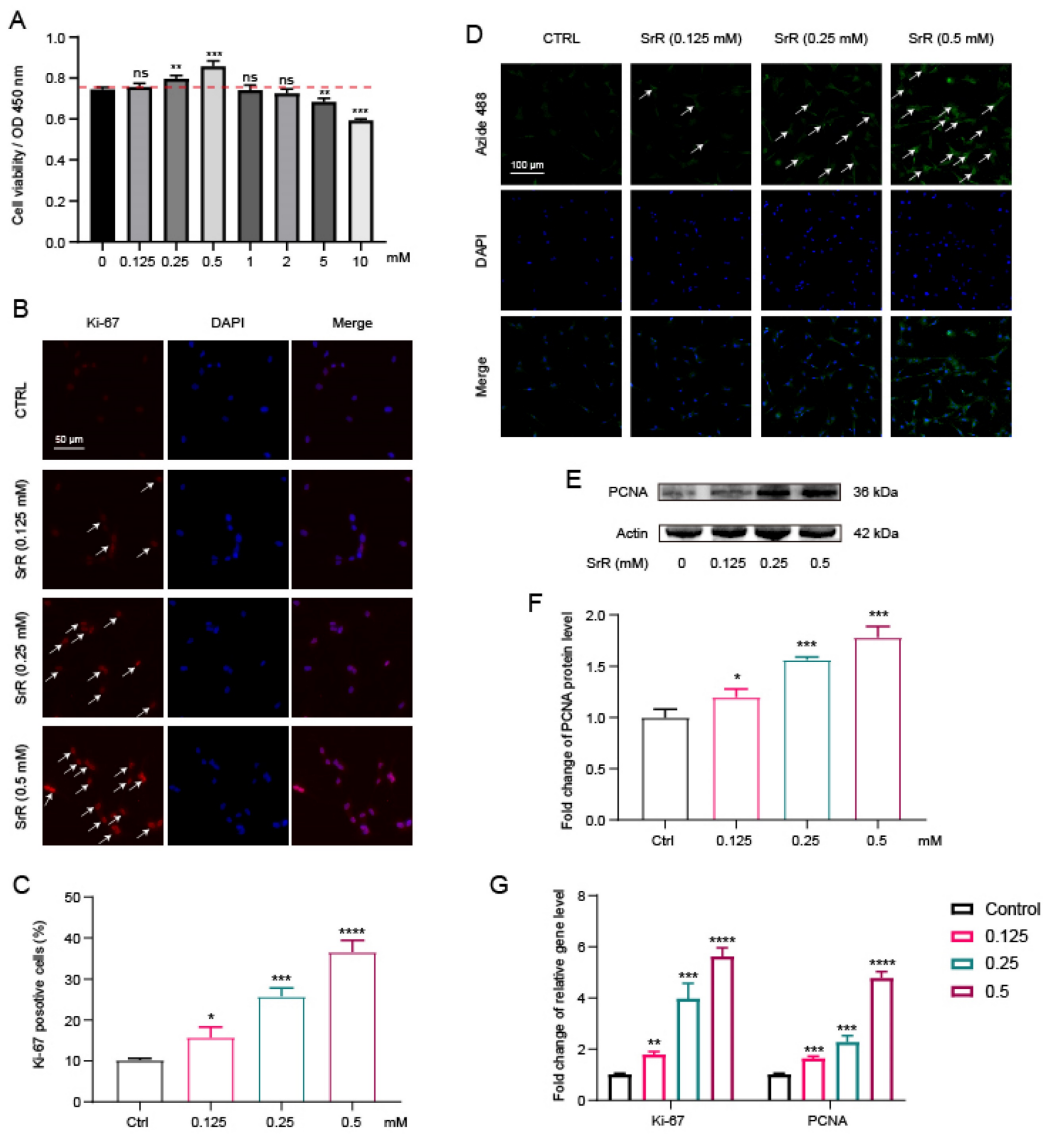
** Effects of SRR on the proliferative ability in human NP cells.** (A)The biological effect of SRR on the cell viability of NP cells (n = 5). (B,C) Expression of Ki-67(green) was detected by the immunofluorescence. The ratios of cells with green fluorescence were calculated. Scar bar = 200 μm. (D) The Alexa Fluor 488 Cell Proliferation Assay Kit examines the effect of different concentrations of SRR (0.125, 0.25, 0.5 mM) on cell proliferation. (E,F) Protein bands and quantification of expression levels of PCNA (n = 3). (G) The qPCR results further confirmed the expression of Ki-67 and PCNA after SRR treatment. ∗p < 0:05, ∗∗p < 0:01, ∗∗∗p < 0:001, ∗∗∗∗p < 0:0001.

To assess the direct biological impact of SRR on HNP cells, various concentrations of SRR (0, 0.125, 0.25, 0.5, 1, 2, 5, 10 mM) were administered to HNP cells, followed by cell viability evaluation using the CCK8 assay. Results demonstrated that SRR significantly enhanced HNP cell viability at concentrations of 0.25 mM and 0.5 mM without evident cytotoxicity at concentrations below 10 mM (Figure 2A). Moreover, both Proliferating Cell Nuclear Antigen (PCNA) and Ki-67 are established markers for cell proliferation. immunofluorescence analysis for Ki-67, a cell proliferation marker, further confirmed the promotional effect of SRR on HNP cell proliferation (Figures 2B and 2C). In Figure 2D, the Alexa Fluor 488 cell proliferation assay kit was used to examine the impact of various SRR concentrations (0.125, 0.25, 0.5 mM) on cell proliferation, and the results indicated that increasing SRR concentrations effectively promoted cell proliferation. Western blot analysis suggested that SRR also enhanced PCNA expression in NP cells in a concentration-dependent manner (Figure 2E). The results of the WB were quantified and standardized in Figure 2F. The qPCR results for Ki-67 and PCNA further supported the increased expression of Ki-67 and decreased expression of PCNA after SRR treatment, which was consistent with the findings above (Figure 2G). In conclusion, these results revealed that SRR enhances HNP cell viability and promotes NP cell proliferation.

### 3.3. SRR Ameliorated ECM Degradation induced by IL-1β in HNP Cells

**Figure 3:**
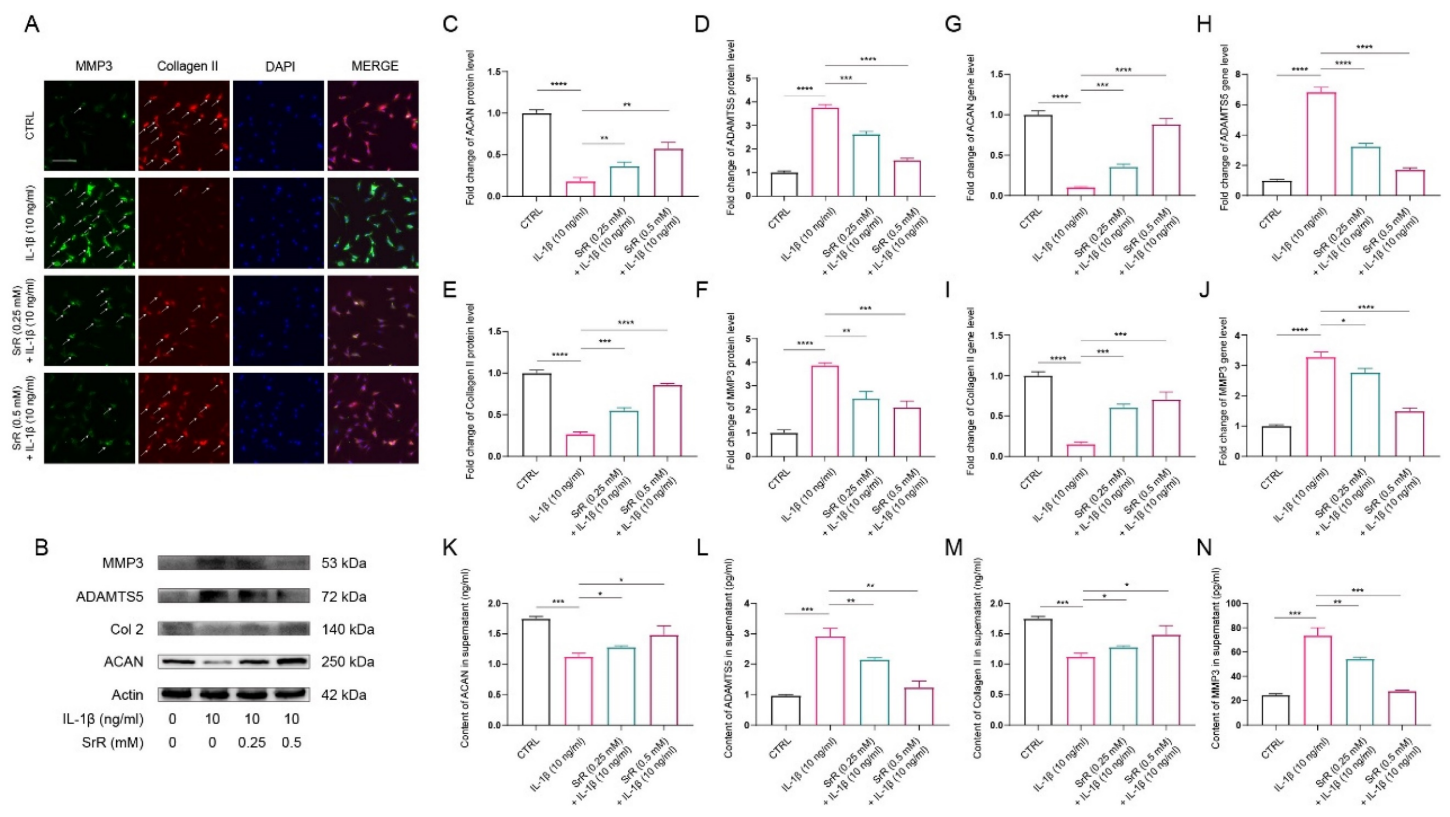
** SRR ameliorated IL-1β-induced ECM degradation in human NP cells.** (A) The expression of MMP3 and type II collagen by the immunofluorescence. Scar bar = 50 μm. (B-F) Protein bands and quantification of protein levels of aggrecan, ADMTS5, type II collagen, and MMP 3. Actin as an internal control (n = 3). (G-J) qRT-qPCR was used to evaluate the mRNA expression of ACAN, ADMTS5, type II collagen, and MMP3 (n = 3). (K-N) IL-1β-induced differential expression of ACAN, ADMTS5, type II collagen, and MMP3 were measured by ELISA cotreated with SRR in a dose-dependent manner in the cultural supernatant of NP cells (n = 3). ∗p < 0:05, ∗∗p < 0:01, ∗∗∗p < 0:001, ∗∗∗∗p < 0:0001.

**Figure 4:**
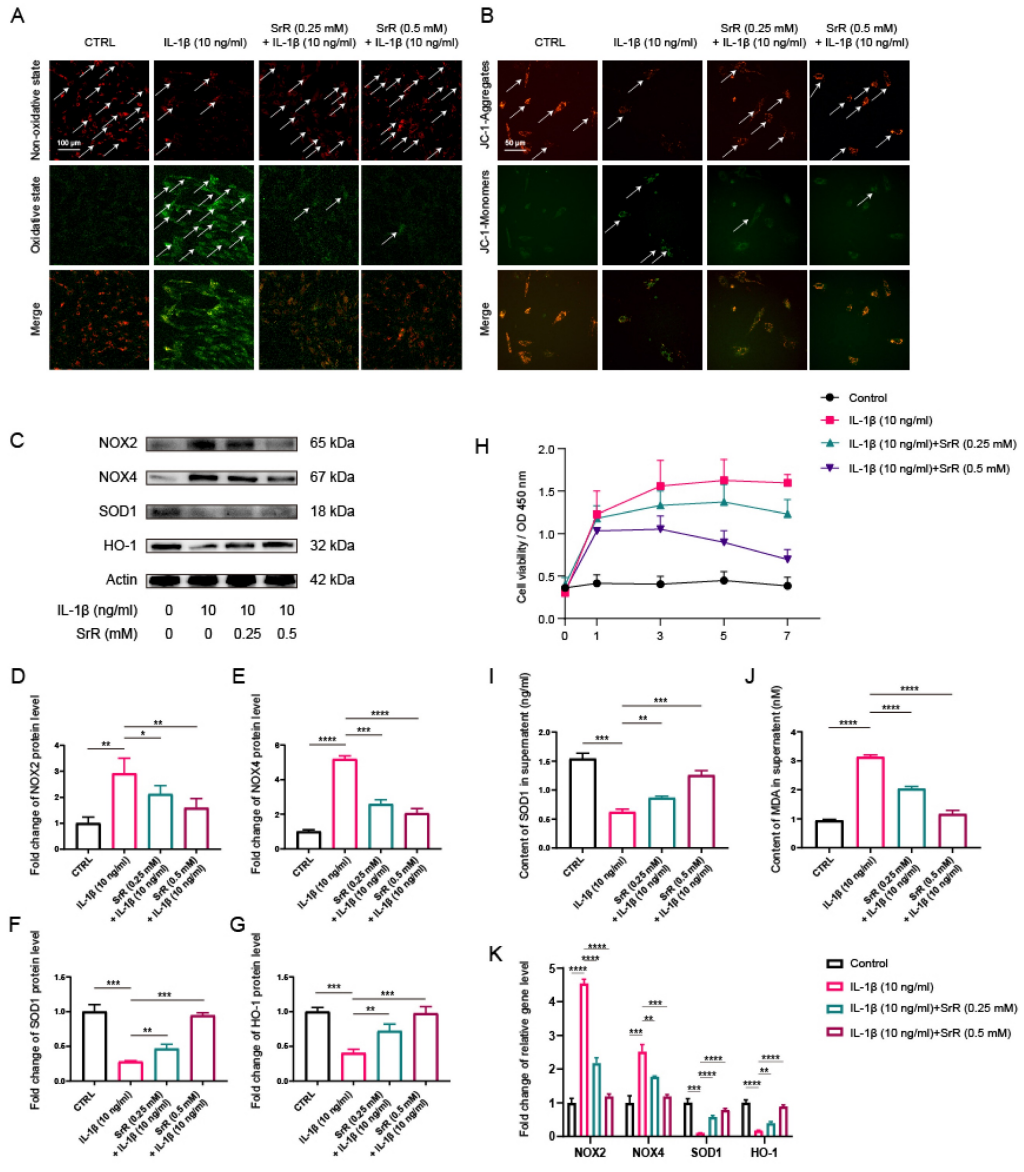
** Effect of SRR on IL-1β-induced oxidative stress in human NP cells.** (A) The feature of BODIPY assay is that the higher the green fluorescence intensity, the higher the lipid peroxidation level. Thus, as presented, the level of lipid peroxidation was much higher in IL-1β group, which could be reversed by SRR. However, the anti-ferroptosis effect of SRR did not show statistically different. (B) The JC-1 monomers (red) and JC-1 aggregates (green) were detected by the fluorescent probe JC-1, and the ratios of JC-1 aggregate/monomer were calculated. Scar bar = 200 μm. (C-G) Protein bands and quantification of protein levels of NOX2, NOX4, SOD1 and HO-1 (n = 3). (H) The Superoxide Assay Kit showed the effect of various concentrations of SRR (0.25,0.5 mM) on cell viability OD under IL-β1-mediated conditions (I, J) IL-1β-induced differential levels of SOD and MDA were assessed by ELISA with SRR in a dose-dependent manner in human NP cells (n = 5). (K) qPCR showed the effect of various concentrations of SRR (0.25, 0.5mM) on NOX2, NOX4, SOD1 and HO-1 gene expression mediated by IL-1β. ∗p < 0:05, ∗∗p < 0:01, ∗∗∗p < 0:001, ∗∗∗∗p < 0:0001.

IVDD is characterized by ECM degeneration. Specifically, a decrease in aggrecan and type II collagen, which are positive markers for ECM integrity, is observed. Conversely, an increase in A Disintegrin And Metalloproteinase with Thrombospondin Motifs 5 (ADAMTS5) and Matrix Metalloproteinase 3 (MMP3), serving as negative markers indicative of ECM degradation, is noted in IVDD^31^. In addition, IL-1β has been shown to play a crucial role in the initiation and progression of IVDD through activating oxidative stress and inflammatory response^32^,^33^. Therefore, we further investigated the effect of SRR on IL-1β-induced HNP cell dysfunction by examining ECM changes. As depicted in Figure 3, immunofluorescence analysis for type II collagen and MMP3 indicated that IL-1β stimulation significantly suppressed type II collagen expression in HNP cells while increasing MMP3 production (Figure 3A). However, these abnormal alterations in the NP cell ECM induced by IL-1β were notably ameliorated by SRR (Figure 3A). As illustrated in Figure 3B, the expression of type II collagen and aggrecan in HNP cells treated with IL-1β (10ng/ml) was markedly suppressed, while MMP3 and ADMTS5 production increased. The results of the Western blot were quantified and standardized in Figures 3C-3F, demonstrating that SRR could reverse the IL-β-induced ECM degradation. The qPCR assay results showed the same trend (Figures 3G-3J). To investigate ECM changes by phenotype, in addition to the aforementioned experiments, we also examined the secretion of catabolic proteinases in IL-1β-induced NP cells via ELISA. The results revealed that pretreatment with SRR could mitigate the increased expression of MMP3 and ADMTS5 induced by IL-1β in a concentration-dependent manner, and that the protein expression of collagen type II and aggrecan was also enhanced by SRR while NP cells were injured by IL-1β (Figures 4K-4N). In conclusion, SRR could ameliorate IL-1β-induced ECM degeneration *in vitro*.

### 3.4. SRR Improved Oxidative Stress Damage in HNP Cells Induced by IL-1β

SRR exhibited anti-oxidative stress effects in HNP Cells induced by IL-1β. An increasing number of studies have demonstrated that aberrant activation of oxidative stress injury is involved in the initiation and exacerbation of IVDD. Furthermore, previous studies have reported that IL-1β acts as a potent activator of oxidative stress injury 32,33. In the present study, IL-1β at a concentration of 10 ng/ml was used to establish the HNP cell injury model. To investigate the effects of SRR on HNP cells under oxidative stress conditions induced by IL-1β, 10 ng/l IL‑1β was applied to chondrocyte culture medium and the cells treated with or without SRR for 1-7 days. The BODIPY assay results indicated that lipid peroxidation levels were significantly increased in HNP cells exposed to IL-1β (Figure 4A). These findings confirmed that the oxidative stress inducer IL-1β could promote HNP cell death, which could be reversed by SRR. Changes in JC-1 staining demonstrated that the mitochondrial membrane potential (MMPo) compromised by IL-1β could be reversed by SRR (Figure 4B). At the molecular level, Western blot analysis demonstrated that NADPH oxidase 2 and NADPH oxidase 4 (NOX2/4), serving as positive markers for cellular oxidative stress, were upregulated in response to IL-1β. Conversely, Superoxide Dismutase 1 (SOD1) and Heme oxygenase-1 (HO-1), both recognized as positive markers for antioxidative defense, exhibited decreased expression. Nevertheless, these effects were all reversed by SRR in a dose-dependent manner (Figures 4C). Superoxide levels increased with the duration of IL-1β treatment, and lower superoxide levels were observed in the SRR group at each time point (Fig. 4D). The results of the Western blot were quantified and standardized in Figures 4(E) to 4(H), demonstrating that SRR could inhibit oxidative stress-related factors NOX2/4 and promote SOD1 and HO-1. ELISA analysis also suggested the suppressive effect of SRR on IL-1β-induced oxidative stress (Figure 4I and 4J). In addition, qPCR results indicated that the pretreatment of the SRR group exhibited lower pro-oxidative stress genes (NOX2 and NOX4) and increased expression of anti-oxidative stress genes (SOD-1 and HO-1) with statistical significance, compared with the IL-1β group (Figure 4K).

### 3.5. SRR Exerted Anti-Inflammatory Effects by Regulating Inflammation-Related Mediators in IL-1β-Induced HNP Cells

**Figure 5:**
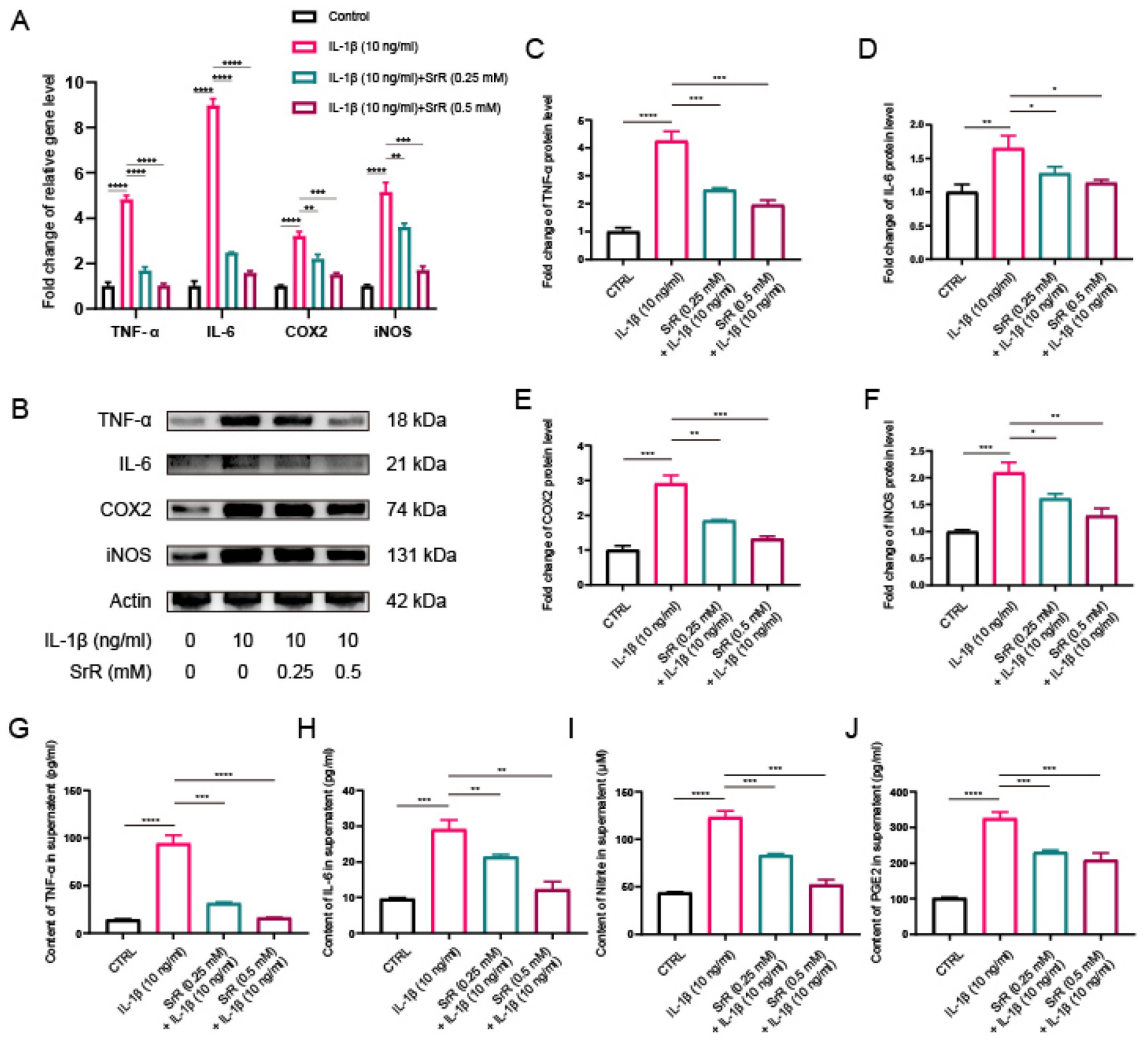
** Impact of SRR on IL-1β-induced expression of inflammatory factors in human NP cells.** (A) qPCR was found to show a significant increase in IL-1β-mediated SRR at various concentrations (0.25,0.5mM) on the expression of TNF-α, IL-6, COX2 and iNOS genes. (B-F) Protein bands and quantification of protein expression of TNF-α, IL-6, COX2 and iNOS. Actin as an internal control (n=3). (G-J) Differential expression of TNF-α, IL-6, Nitrite, and PGE2 were quantified by ELISA assay (n = 5). ∗p < 0:05, ∗∗p < 0:01, ∗∗∗p < 0:001, ∗∗∗∗p < 0:0001.

During IVDD, excessive focal inflammatory responses can impair the normal biological function of NP cells in a positive-feedback manner. Consequently, inhibiting the abnormal inflammatory microenvironment in disc tissue has become a therapeutic target 32,33. Thus, we further explored the impact of SRR on the IL-1β-induced inflammatory response. Cyclooxygenase-2(COX-2), inducible Nitric Oxide Synthase (iNOS), Interleukin-6 (IL-6), Nitrite, Prostaglandin E2 (PGE2) and Tumor Necrosis Factor-alpha (TNF-α) are all established markers for inflammation. Besides, COX-2 and iNOS have been previously reported as two key inflammatory mediators during IVDD. In the present study, we initially examined the protein levels of COX2 and iNOS in NP cells treated with IL-1β, with or without SRR. Previous studies have revealed that iNOS overexpression promotes NO production, which then stimulates COX2 activation^34^. Therefore, we further investigated the effects of SRR on the IL-1β-induced inflammatory response in HNP cells. As demonstrated in the results, the expression of proinflammatory genes IL-6, TNF-α, COX2, and iNOS were significantly suppressed by SRR (Figure 5A). At the molecular level, the inhibitory effect of SRR on proinflammatory protein expression in HNP cells was confirmed by Western blotting (Figure 5B). The results of Western blotting were quantified in (Figure 5C-5F). Additionally, ELISA results revealed that Nitrite and PGE2 production in HNP cells dramatically increased following IL-1β treatment; however, this effect was reversed by SRR (Figure 5G-5J). In conclusion, the findings indicated that SRR could alleviate the IL-1β-induced inflammatory response in HNP cells *in vitro*.

### 3.6. SRR Regulated IL-1β-Induced NF-κB Activation in NP Cells

**Figure 6:**
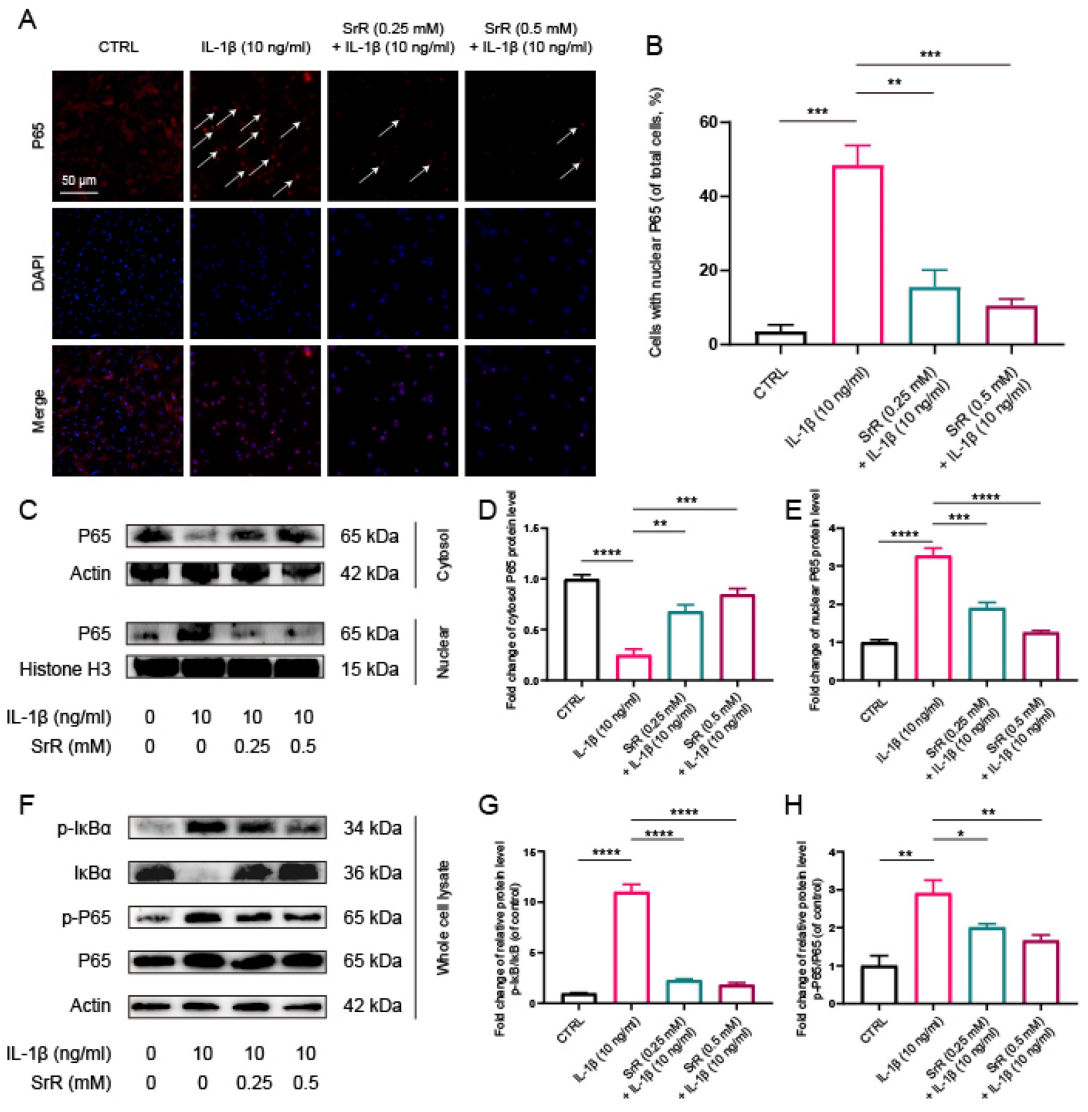
** Effects of SRR on the activation of NF-κB pathway induced by IL-1β in human NP cells.** (A,B) The nuclei translocation of p65 in different groups was visualized. Intensity of nuclear p65 fluorescence was quantified (n = 3). Scar bar = 200 μm. (C-E) Differential expressions of p65 in cytoplasm and in nucleus were visualized and quantified by WB, respectively (n = 3). (F-H) Protein levels of IκBα, p65, and their phosphorylated forms were analyzed by WB in different groups (n = 3). ∗p < 0:05, ∗∗p < 0:01, ∗∗∗p < 0:001, ∗∗∗∗p < 0:0001.

SRR was found to suppress IL-1β-induced activation of the NF-κB signaling pathway in HNP cells. NF-κB, P65, and IκB Kinase-beta (IKK-β) serve as key positive regulators in cellular stress and inflammatory responses, whereas the Inhibitor of kappa B (IκB) family proteins, particularly Inhibitor of kappa B alpha (IκB-α), act as negative regulators of NF-κB activity. Numerous studies have demonstrated that the NF-κB signaling pathway plays a role in the initiation and exacerbation of IVDD by promoting ROS generation and inflammatory responses^24^,^35^. In the control group, P65, the main component of NF-κB, was predominantly detected in the cytoplasm. However, following IL-1β treatment, P65 nuclear translocation in HNP cells was significantly increased, which was dramatically reversed by SRR [Figure 6A, 6B]. Moreover, the inhibition of P65 nuclear translocation, as evidenced by Western blot results, was consistent with immunofluorescence findings (Figure 6C-6E). Generally, NF-κB exists in an inactive state in the cytoplasm due to its interaction with IκB family proteins, which prevent NF-κB nuclear translocation. Upon stimulation, phosphorylated IKK-β activates IκB-α, leading to its phosphorylation and subsequent degradation, allowing NF-κB to be released and translocated into the cell nucleus^36^. Consequently, we also investigated changes in IκB-α and discovered that IL-1β treatment significantly promoted the phosphorylation and degradation of IκBα in the cytoplasm. The increased amount of P65 in the cytoplasm of SRR-treated NP cells further confirmed the suppression of IL-1β-activated nuclear translocation of P65 (Figures 6F-6H). In conclusion, SRR inhibited IL-1β-induced activation of NF-κB by preventing IκBα phosphorylation in the cytoplasm, thus suppressing P65 translocation into the nuclei of NP cells.

### 3.7. Molecular docking predicted an interaction between SRR and TGF-β1

**Figure 7:**
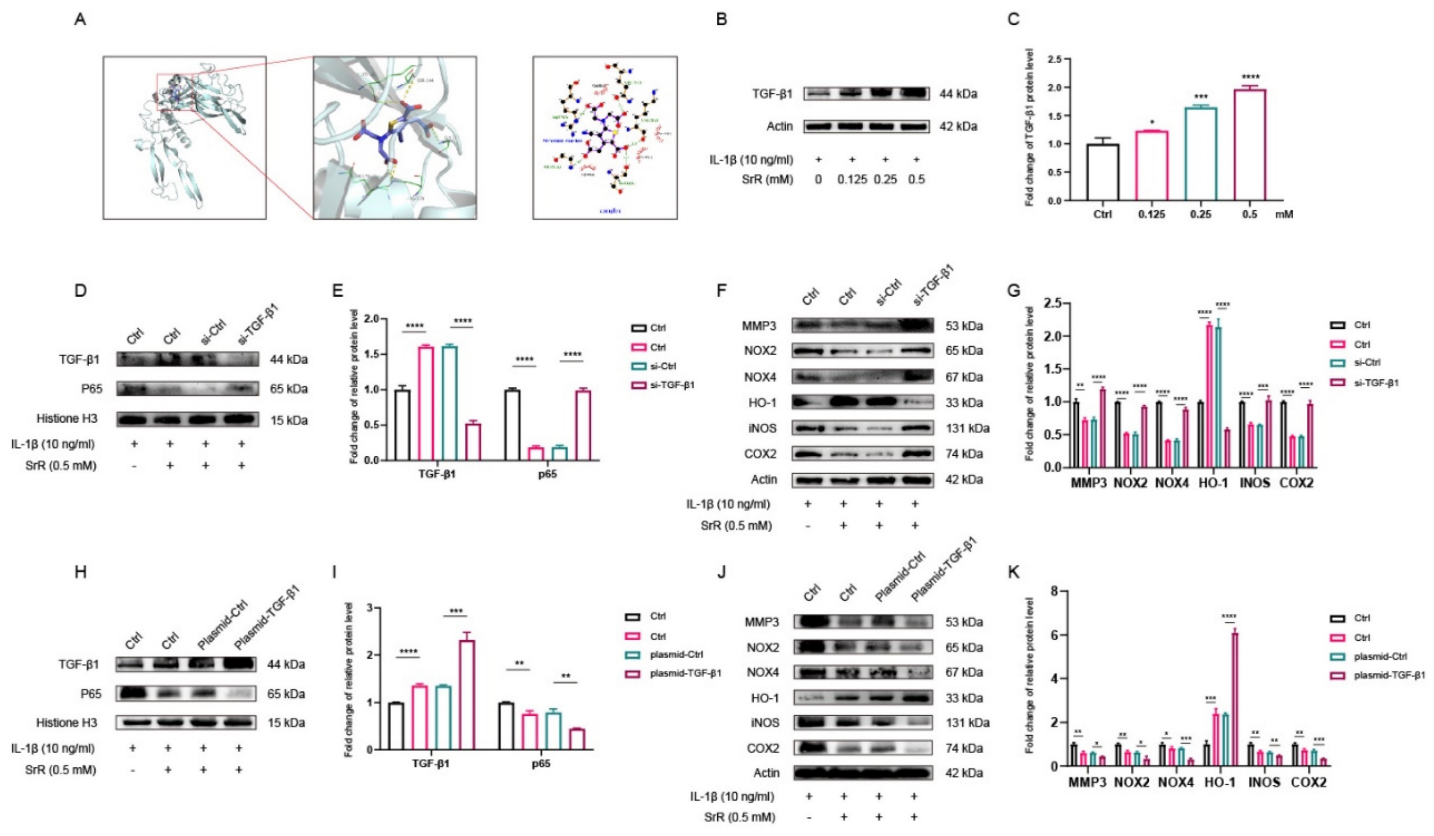
** SRR Modulates IL-1β-Induced Apoptosis, Inflammatory Response, and Oxidative Stress via TGF-β1 Signaling Pathway in Human NP Cells.** (A) The result showed the overall binding view and local binding view of TGF-β1-SRR complex obtained based on docking, where the yellow dotted line represents the hydrogen bond interaction, and the green line represents the amino acid where the binding site forms hydrogen bond with SRR. (B,C) WB indicated that SRR could promote the expression of TGF-β1 in a dosedependent manner. (D,E) The WB showed that siRNA-TGF-β1significantly inhibited the TGF-β1expression compared with siRNA-Ctrl. (F,G) The expression of MMP3, NOX2, NOX4, iNOS and COX2 was upregulated after the HNP cells were pre-transfected with siRNA-TGF-β1. However, the absence of TGF-β1 compromised the expression of HO-1. Collectively, TGF-β1 played the pivotal role in ameliorating ECM degradation and oxidative stress, and the biological effects of SRR on HNP cells were TGF-β1-dependent. (H,I) The WB showed that Plasmid-TGF-β1significantly prmoted the TGF-β1expression compared with Plasmid-Ctrl. (J,K) After pre-transfection of HNP cells with Plasmid-TGF-β1 promoted HO-1 expression, however, Plasmid-TGF-β1 resulted in the suppression of MMP3, NOX2, NOX4, iNOS and COX2 expression. ∗p < 0:05, ∗∗p < 0:01, ∗∗∗p < 0:001, ∗∗∗∗p < 0:0001.

Docking simulation techniques have been a convenient and effective means of probing the interaction of small molecules with their target targets. In the current study, the small molecule Strontium ranelate was docked to the TGF-β1 protein using AutoDock Vina 1.1.2 software. Negative binding energies indicate the possibility of binding, and values less than -5 kcal/mol are usually considered to have a higher binding potential. In this study, the binding affinity of the small molecule Strontium ranelate to the TGF-β1 protein was below -5 kcal/mol, which means that the small molecule Strontium ranelate has a more desirable potential activity effect with the TGF-β1 protein. As shown in Figure 7A, where the yellow dashed line represents the hydrogen bond, the green line indicates the amino acid that forms a hydrogen bond with the small molecule Strontium ranelate in the protein binding pocket, the cartoon indicates the TGF-β1 protein and the purple stick indicates the Strontium ranelate molecule. In the TGF-β1-Strontium ranelate complex, the small molecule Strontium ranelate is bound to the TGF-β1 protein in a pocket surrounded by amino acids GLN141, TYR143, GLU184, GLY180, ARG179, GLN175, LYS142, SER144 and GLY181. Among the pockets formed by GLN141, TYR143, GLU184 and GLY180, which form hydrophobic interactions. Taken together, the main interaction of the TGF-β1 protein with the small molecule Strontium ranelate is hydrogen bonding and hydrophobic interaction, which is likely to be the main type of effect of the small molecule Strontium ranelate on the TGF-β1 protein.

### 3.8. SRR Modulates IL-1β-Induced Apoptosis, Inflammatory Response, and Oxidative Stress via TGF-β1/NF-κB Signaling Pathway in HNP Cells

As demonstrated, Western blot results indicated that IL-1β downregulated TGF-β1 levels in HNP cells, while these effects were attenuated by SRR (Figures 7A and C). To further investigate the pivotal role of TGF-β1 in the impact of SRR on HNP cells, a TGF-β1 knockdown model was established using siRNA-TGF-β1. SiRNA-TGF-β1 significantly inhibited TGF-β1 expression, confirming the successful establishment of the TGF-β1 knockdown model (Figures 7D and 7E). Furthermore, the expression of nuclear P65 in the siRNA-TGF-β1 group was significantly increased compared to the siRNA-Ctrl group following IL-1β stimulation, with or without SRR administration. Additionally, after HNP cells were pre-transfected with siRNA-TGF-β1, the expression of MMP3, NOX2/4, iNOS, and COX2 was upregulated, implying that the absence of TGF-β1 might exacerbate ECM degradation (Figure 7F). Conversely, the expression of HO-1 was considerably lower in the siRNA-TGF-β1 group compared to the siRNA-Ctrl group, highlighting the essential role of TGF-β1 in the anti-oxidative stress effect (Figure 7F). To further explore the key function of TGF-β1 in the impact of SRR on HNP cells, a TGF-β1 overexpression model was established using a plasmid encoding TGF-β1 (plasmid-TGF-β1). As depicted in Figure 7H, TGF-β1 overexpression was evident, verifying the successful establishment of the TGF-β1 overexpression model. Moreover, nuclear P65 expression in the plasmid-TGF-β1 group was significantly reduced compared to the control group after IL-1β stimulation, regardless of SRR treatment (Figure 7I). Additionally, when HNP cells were transfected with plasmid-TGF-β1, the expression of MMP3, NOX2/4, iNOS, and COX2 was downregulated, suggesting that the presence of TGF-β1 might alleviate ECM degradation (Figure 7J). In contrast, the expression of HO-1 was significantly higher in the plasmid-TGF-β1 group compared to the control group, indicating the crucial role of TGF-β1 in the anti-oxidative stress effect (Figure 7K).

### 3.9. Evaluating the Protective Effects of SRR on Puncture-Induced IVDD In vivo

**Figure 8:**
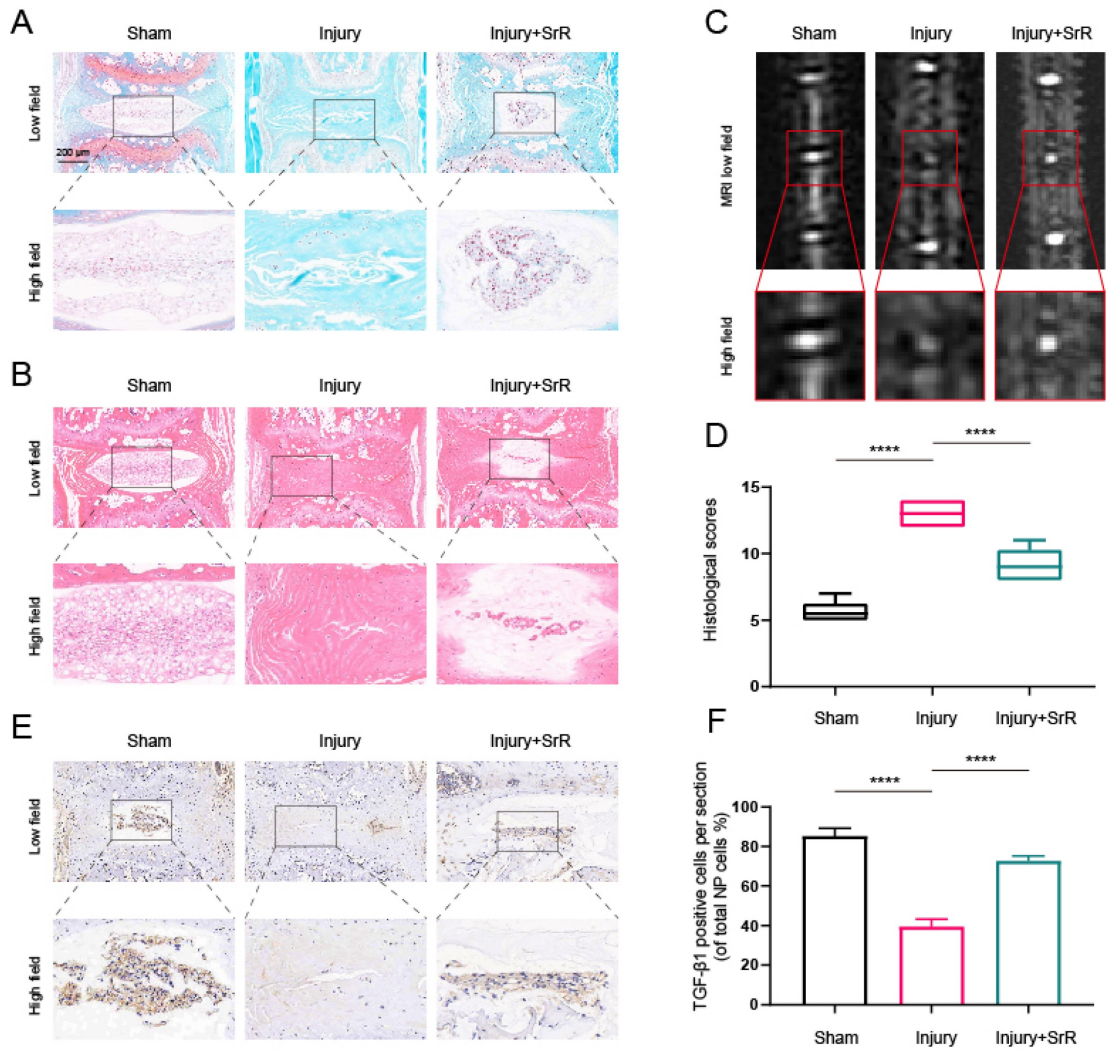
** Potential of SRR to mitigate IVDD progression *in vivo*.** (A,B) Selected H&E staining and S-O staining images of punctured intervertebral discs across different groups. The group subjected to injury revealed substantial AF rupture, reduced NP cell count, and diminished proteoglycan levels compared to the sham surgery group, all of which were partially mitigated by SRR (500 mg/kg/day). (C) T2-Weighted MRI image of a mouse tail captured at the 8th week post-surgery. (D) The assessment of histological grades was carried out. (E,F) IHC targeting TGF-β1 demonstrated that SRR (500 mg/kg/day) has the potential to elevate the expression of TGF-β1. ∗p < 0:05, ∗∗p < 0:01, ∗∗∗p < 0:001, ∗∗∗∗p < 0:0001.

The present investigation further evaluated the potential of SRR in mitigating the damage induced by injury in IVDD *in vivo*. An IVDD mouse model was established through a puncture method and the morphological alterations of the disc were studied using Hematoxylin and Eosin (HE) and Safranin O (SO) staining methods. The injury group, in comparison with the sham surgery group, demonstrated a severe rupture in the annulus fibrosus (AF), a diminished count of NP cells, and a loss of proteoglycans. The histological grading was conducted based on the methodologies reported in an earlier study^37^. A score of 5 was given for normal discs, 6-11 for moderately degenerated discs, and 12-14 for severely degenerated discs. The application of SRR was found to mitigate these histopathological alterations, as evidenced by lesser loss of NP cells and proteoglycans (Figure 8A, B and D). Further analysis of IVDD severity was performed via MRI after 8 weeks of treatment. It was observed that the injury group displayed a signal loss in MRI images, a trend that could be partly reversed with the introduction of SRR (500 mg/kg/day) (Figure 8C). Furthermore, SRR (500 mg/kg/day) administration was found to ameliorate the suppressed expression of TGF-β1 triggered by IVDD, as suggested by the increased TGF-β1 expression (Figure 8E and F). In summary, SRR demonstrated potential for delaying the IVDD progression *in vivo*.

## 4. Discussion

With the advent of the fourth industrial revolution and the increased proportion of sedentary work in human lives, it is estimated that up to 80% of individuals will experience low back pain (LBP) during their lifetimes^38^. Alarmingly, the incidence of LBP is rising at an unprecedented rate among young adults^30^. Research has shown that IVDD is a primary contributor and independent risk factor for LBP, highlighting the importance of investigating the exact pathogenesis of IVDD and developing targeted molecular therapies in the future. Extracellular matrix (ECM) degradation, characterized by elevated expression of matrix metalloproteinases (MMPs) and reduced expression of aggrecan and type II collagen, is a hallmark and major pathological feature of IVDD, ultimately resulting in protein denaturation, necrosis, disruption of NP cells metabolic homeostasis, and IVDD^39^. The upregulation of MMPs (such as MMP3, MMP13, etc.) can lead to abnormal matrix degradation, loss of disc height, and biomechanical dysfunction of IVDD^40^. Our study revealed that SRR effectively ameliorated IL-1β-induced ECM degradation by upregulating the expression of type II collagen and aggrecan and downregulating MMP3 and ADMTS5. These findings are consistent with previous studies that have reported the chondroprotective effects of SRR in osteoarthritis^41^. This suggests that SRR might exert a protective effect on IVDD by preserving the ECM composition and structure, thereby maintaining disc biomechanical function.

In addition, the current study suggested that SRR significantly augmented HNP cell viability, thereby endorsing its potential therapeutic role in the management of IVDD. Past studies have demonstrated the value-added promoting effects of SRR on other cell types, which include muscle cells, nerve cells and tumor cells. However, this is the first report that SRR can promote the value-added of myeloid cells, which offers a new potential strategy for the treatment of IVDD. Cell proliferation is a fundamental process in maintaining tissue homeostasis. In the context of IVDD, the preservation and enhancement of cell proliferation is paramount, particularly within the NP. The NP comprises a dense population of cells that provide essential support to the IVDD, contributing to its structural integrity and function. It has been widely recognized that the diminution in the quantity of NP cells is a critical juncture in the pathogenesis of IVDD^42^-^44^.Emerging studies have indicated that the SRR treatment may not only promote NP cell proliferation but also possibly enhance the overall cell population within the IVDD. By stimulating cell proliferation, SRR could potentially address the cell deficit observed in IVDD, thereby contributing to the restoration of IVDD integrity. The connection between the decline in NP cell numbers and IVDD progression underscores the necessity for therapies that can effectively boost NP cell proliferation. Given the apparent role of SRR in stimulating this process, it provides a promising prospect in the treatment or possible reversal of IVDD. The potential of SRR to act as a therapeutic agent may extend beyond just restoring the NP cell numbers; it could also impact the broader physiological landscape of the IVDD, thereby addressing the multifaceted nature of IVDD. However, it is essential to note that our understanding of the mechanisms through which SRR enhances cell proliferation is still evolving. The precise pathways involved, the optimal dosages and treatment durations for achieving maximum therapeutic efficacy, and the potential side-effects, all require comprehensive investigation in future studies. As we advance our knowledge of SRR's impacts on cell proliferation, we can fine-tune its application, maximizing its potential as an effective therapeutic agent in the battle against IVDD.

The intricate relationship between oxidative stress and inflammation in the pathogenesis of IVDD has been well established. This relationship is symbiotic, multifaceted, and complex; oxidative stress can induce an inflammatory response that, in turn, intensifies the oxidative stress damage^45^. Specifically, oxidative stress triggers a cascade of inflammatory responses by activating NF-κB signaling pathway, a master regulator of inflammatory and immune responses^46^-^48^. Concurrently, the activated inflammatory response produces Reactive Oxygen Species (ROS) thereby enhancing oxidative stress injury. Moreover, there's a reciprocal regulation between oxidative stress and inflammation via shared signaling pathways such as MAPK and PI3K/AKT, leading to the perpetuation of a vicious cycle that progressively exacerbates IVDD^49^. Hence, it's crucial to highlight that this isn't a unidirectional relationship but a mutual amplification and exacerbation, making it a key target for potential therapeutic interventions. Inflammatory cytokines-induced oxidative stress can directly or indirectly accelerate the inflammatory response, further activating additional oxidative stress^50^. Oxidative stress and inflammation are known to contribute to the initiation and progression of IVDD^45^,^50^. In our study, SRR exhibited potent antioxidative and anti-inflammatory effects in IL-1β-stimulated HNP cells. SRR treatment significantly reversed the upregulation of pro-oxidative stress-related proteins (NOX2/4) and proinflammatory mediators (COX2, iNOS, IL-6, and TNF-α) induced by IL-1β. Moreover, SRR also enhanced the expression of antioxidative stress-related proteins (SOD1 and HO-1) and alleviated the IL-1β-induced inflammatory response. These results further highlight the potential of SRR in mitigating the detrimental effects of oxidative stress and inflammation during IVDD.

Our findings demonstrated that SRR could ameliorate IL-1β-induced apoptosis, inflammatory response, oxidative stress, and ECM degradation in HNP cells. In this study, we also investigated the underlying molecular mechanisms of protective effects of SRR on IVDD, and moreover, our molecular docking results revealed a potential interaction between SRR and TGF-β1 protein. These findings provide novel insights into the therapeutic potential of SRR for IVDD treatment. Firstly, we observed that the expression of TGF-β1 decreased, while the expression of NF-κB and IL-1β increased in degenerated human disc tissue samples. This observation is consistent with previous studies, which have shown that the dysregulation of NF-κB is associated with IVDD progression and severity 50-52. Thus, we hypothesized a potential link between TGF-β1 and the NF-κB signaling pathway in the context of SRR-mediated protection against IL-1β-induced cellular stress in NP cells. As shown in the molecular docking results, we proved the interaction between SRR and TGF-β1 protein. To verify this interaction, gain- and loss- of function experiments were conducted. We discovered that both TGF-β1 knockdown and overexpression influenced the expression of nuclear P65, a pivotal component of the NF-κB pathway^51^. The NF-κB pathway is a key cell signaling system involved in numerous cellular processes, including inflammatory responses, cell growth, and survival. Under normal conditions, NF-κB proteins are kept inactive in the cytoplasm through their interaction with inhibitory IκB proteins. Upon receiving signals such as those from cytokines, growth factors, or stress, IκB proteins become phosphorylated and subsequently degraded, allowing the release and nuclear translocation of active NF-κB^52^. Once in the nucleus, NF-κB can bind to specific sequences of DNA, leading to the transcription of target genes. The modulation of nuclear P65 expression by TGF-β1 suggests that TGF-β1 might participate in the activation and regulation of this signaling pathway. Specifically, alterations in TGF-β1 levels could potentially influence the stability or degradation of IκB proteins, or affect the nuclear translocation of P65. This indicates a potential role for TGF-β1 as a regulatory factor in this signaling cascade. This is in line with previous studies showing that TGF-β1 can inhibit the activation of the NF-κB pathway in various cell types, including chondrocytes and osteoblasts^53^. Our results, therefore, suggest that SRR may exert its protective effects on NP cells by modulating the TGF-β1/NF-κB signaling axis. However, further studies are needed to elucidate the precise molecular mechanisms underlying this interaction and to determine whether other signaling pathways may also be involved in SRR-mediated protection against IVDD.

In the current investigation, we successfully applied the well-established puncture-induced model of IVDD. This technique is an accepted method in the field due to its efficiency and accuracy in mimicking the degenerative process of human IVDD, which is crucial for the development and assessment of potential therapeutic strategies. Our findings confirmed that the puncture method indeed leads to significant inflammatory damage within the intervertebral discs (IVDs), which mimics the pathological changes observed in human degenerative disc disease.

In this context, we further explored the potential therapeutic implications of SRR on IVDD through a series of *in vivo* experiments. As an innovative approach in IVDD research, this study aimed to leverage the unique pharmacological properties of SRR, particularly its dual action in promoting bone formation and inhibiting bone resorption, which could have promising applications in IVDD treatment. Encouragingly, the* in vivo* findings demonstrated that SRR administration could effectively alleviate the IVDD caused by the puncture. This outcome was most notably observed in the attenuation of morphological changes commonly associated with IVDD, such as the loss of disc height and disorganization of the annulus fibrosus structure. Importantly, SRR treatment was shown to preserve disc height, suggesting its potential in maintaining the structural integrity of IVDs. These results are particularly significant when considered in the light of SRR's known capabilities in bone health and disease. The beneficial effects of SRR in maintaining disc height could be attributed to its known mechanisms of promoting bone formation while inhibiting bone resorption, which may help preserve the structural integrity of degenerating discs.

In conclusion, our study demonstrates that SRR effectively ameliorates IL-1β-induced apoptosis, inflammatory response, oxidative stress, and ECM degradation in HNP cells by modulating the TGF-β1/NF-κB signaling pathway. The potential interaction between SRR and TGF-β1 protein suggests a novel molecular mechanism for the protective effects of SRR against IVDD. These findings provide valuable insights into the therapeutic potential of SRR for the treatment of IVDD and pave the way for further in-depth research on its clinical application.

## Limitations

Despite the promising results of our study, there are several limitations that should be acknowledged. Firstly, our study was mainly conducted *in vitro* using HNP cells, which may not fully recapitulate the complex *in vivo* environment of the intervertebral disc. Future studies using animal models of IVDD and ultimately clinical trials are necessary to validate our findings and establish the therapeutic efficacy of SRR in treating IVDD. Secondly, while our molecular docking analysis suggests a potential interaction between SRR and TGF-β1 protein, additional experimental validation, such as surface plasmon resonance or isothermal titration calorimetry, is required to confirm this interaction and elucidate the precise molecular mechanism. Finally, the potential side effects and optimal dosage of SRR for the treatment of IVDD need to be carefully investigated to ensure its safety and efficacy in clinical applications. Further research using *in vivo* models and clinical trials is warranted to validate these findings and facilitate the development of SRR as a therapeutic option for patients suffering from IVDD.

## Conclusion

In summary, this study elucidated the molecular mechanisms through which SRR inhibits IL-1β-mediated disc degeneration by modulating the TGF-β1/NF-κB pathway, using *in vitro* and *in vivo* models. Our findings may contribute to the development of SRR-based therapeutics for the treatment of IVDD and provide valuable insights into the complex interplay between inflammation, disc cell biology, and ECM homeostasis in the pathogenesis of IVDD. Ultimately, our research may pave the way for novel therapeutic strategies targeting the TGF-β1/NF-κB pathway, offering hope for millions of individuals suffering from the debilitating consequences of IVDD.

## Figures and Tables

**Table 1:** Central coordinates of protein and docking box parameters.

Protein	Central coordinates of protein (X, Y, Z)	Parameters of docking box (X, Y, Z)
TGF-β1	80.405, 44.585, 34.318	94.0, 64.0, 96.0
